# Addressing Social Determinants of Health Service Gaps in Chinese American Caregivers During the COVID-19 Pandemic

**DOI:** 10.3390/children12111499

**Published:** 2025-11-05

**Authors:** Alicia Chung, Stella Chong, Debbie Chung, Amira Gee, Monica Stanton-Koko, Keng-Yen Huang

**Affiliations:** 1 Department of Population Health, NYU Grossman School of Medicine, New York, NY 10016, USA; amira.gee@nyulangone.org (A.G.); keng-yen.huang@nyulangone.org (K.-Y.H.); 2 Department of Epidemiology and Biostatistics, City University of New York Graduate School of Public Health & Health Policy, New York, NY 10027, USA; stella.chong28@sphmail.cuny.edu; 3 Department of Psychiatry and Behavioral Sciences, University of Miami Miller School of Medicine, Miami, FL 33136, USA; dxc1729@miami.edu; 4 Health Studies Department, Borough of Manhattan Community College, City University of New York, New York, NY 10007, USA; mstantonkoko@bmcc.cuny.edu

**Keywords:** social determinants of health, immigrant health, Chinese Americans

## Abstract

**Highlights:**

**What are the main findings?**

- Caregiver and provider perspectives are both needed to understand how patients navigate addressing social determinants of health for their children and families.
- Patient cultural values and beliefs are integral to understanding how they contribute to patient behaviors and when designing interventions to address social determinants of health.

**What are the implications of the main findings?**

- Adaptations in health services settings need to consider cultural diversity and language concordance among Chinese American immigrant populations based on their region of origin.
- Structural limitations in health services workflow (e.g., reduced staff, digital tools not in the patient’s primary language) may inhibit caregivers’ ability to address social determinants of health needs efficiently.

**Abstract:**

**Background/Objectives**: This study aims to understand gaps and strategies in Chinese Americans’ utilization of SDOH services in the pediatric primary care context in Sunset Park, Brooklyn, from a patient–provider partnership perspective. **Methods**: The study was guided by an integrated Patient–Provider Partnership, Engagement, and Collaboration (PEC) framework that influenced patient–provider interaction during the provision of SDOH services. A qualitative study design was applied, and eight quality improvement interviews with healthcare providers were conducted to understand the existing community and health service system context. Six in-depth interviews were conducted with Mandarin-speaking Chinese American caregivers. Interviews were transcribed and coded in Mandarin and then translated into English. **Results**: Consistent with the PEC framework, we identified cognitive, affective, and communication gaps from both the patient and provider. Caregivers reported unaddressed needs in food, financial security, and mental health. Providers identified gaps in patient workflow, staffing, and the intake form process. **Conclusions**: Addressing social determinants of health among Chinese American immigrant populations is crucial for mitigating poor health outcomes in children and families. Multi-level community-engaged strategies are needed to alleviate the challenges facing this community. Recommendations for future research should consider the importance of language and cultural affinity, digital intake forms translated into the patient’s language, and regular on-site staffing during SDOH screenings.

## 1. Introduction

Healthy People 2030 defines social determinants of health (SDOH) as the following five domains where people are born, live, work, worship, and play: economic stability, education access and quality, healthcare access and quality, neighborhood and built environment, and social and community context [1]. SDOH are a key determinant of poor health outcomes, especially for low-resourced, racial/ethnic minority populations, with four out of five physicians confirming that unmet social needs are directly linked to poor health [2]. Addressing SDOH is essential to narrowing the health disparities gap [3]. Since the COVID-19 pandemic, many national, federal organizations, and healthcare networks have advocated or mandated health systems to integrate SDOH screening and provide services to address unmet needs [4]. Although there is a good intention to address patient SDOH needs, many providers have failed to actually meet those needs [3]. This is particularly true for marginalized, immigrant, low-resourced communities during the COVID-19 pandemic [5,6,7], particularly among Chinese American patients [8,9]. Our study was designed to identify gaps and potential strategies to improve SDOH screening and patient utilization of SDOH services among Chinese American caregivers of young children. To this end, going forward in this manuscript, patients will refer to individuals who interact with the pediatric primary care provider (or family health center) and who are caregivers of young children, 3–8 years old.

There are many factors affecting patients’ utilization of SDOH services, including provider and patient factors. It is critical to understand perspectives from both sides that can inform future culturally tailored interventions. In Joo and Liu’s 2021 scoping review about culturally tailored interventions for ethnic minorities, the authors identified four weaknesses in culturally tailored interventions: (1) unclear guidelines; (2) low attention and retention rates; (3) failure to measure processes; and (4) inadequate training for healthcare providers [10]. This scoping review provides some directions for examining why the SDOH services are not working. However, this review mainly focuses on providers’ or structural gaps in setting up and implementing SDOH. It did not provide insights from the patients’ perspective.

**Figure 1:**
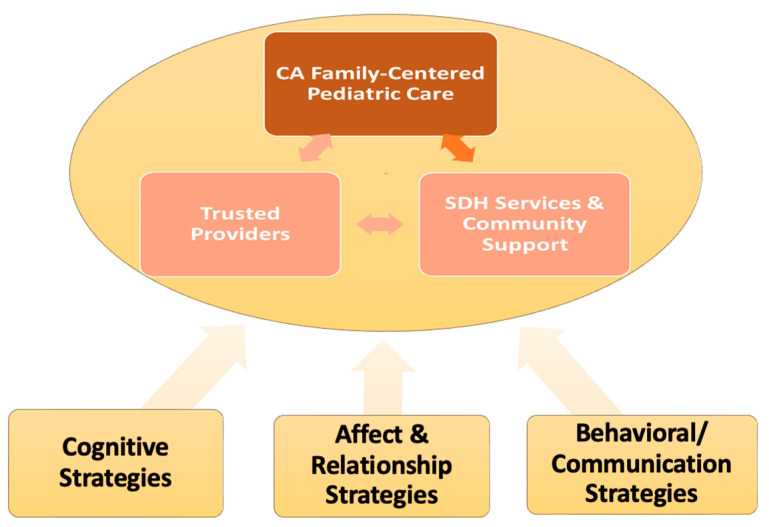
Guiding Framework for Strategy Identification and Development.

To understand gaps in racial and ethnic minority’s utilization of SDOH services, we applied an integrated Partnership, Engagement, and Collaboration (PEC) Framework (see Figure 1), adapted from a multi-level partnership framework [11]. We leveraged this framework, developed by the senior author, YH, to consider the multiple contexts at the socio-political level, as well as the local community and healthcare system that affect how Chinese American caregivers navigate addressing social determinants of health in a post-COVID-19 era. The PEC framework considers contexts/determinants that influence patient–provider interaction and collaboration in gaining SDOH services information, decision making, and service utilization. Our framework considers family-provider partnership as the key to engaging family SDOH services use and adherence. Our conceptual framework considers 4 domains that potentially influence effective family-provider partnerships in addressing patients’ SDOH needs: (1) cognitive (e.g., knowledge; perspectives); (2) affective and interpersonal relationships (e.g., trust, empathy); (3) behavioral/communication (e.g., culturally sensitive communication and actions to increase patient engagement in uptake of evidence-based intervention); and (4) contextual factor domain (e.g., structural or social determinants in primary care setting; individual characteristics) [12].

We carried out a case study that focused on a predominantly Chinese American (CA) immigrant patient population in Sunset Park, Brooklyn, that tends to under-report and under-utilize SDOH screening and services [13]. Nearly 1 million Asian people reside in New York City, with 43% of the Sunset Park population in Brooklyn being Chinese-speaking [14,15]. We focused on Chinese American immigrants because their population growth in New York City is outpacing that of any other minority racial/ethnic groups, and now constitutes almost half of all Chinese Americans living in the northeastern United States. Studies have found that Chinese Americans living in these ethnic enclaves are associated with poorer community health outcomes, such as negative self-perceptions of health, delays in receiving healthcare, low full-time employment and education rates, and high rates of poverty [16]. This spatial segregation, especially in neighborhoods like Sunset Park, reflects broader systemic issues that contribute to the lack of health service utilization and exacerbate health disparities among American residents.

### 1.1. Study Objectives

The objectives of this study are to: (1) identify gaps/barriers in (i.e., Chinese Americans’) screening and utilizing SDOH services in racial/ethnic minorities in the pediatric primary care context; and (2) identify potential strategies to address the barriers. Findings will be used to inform quality improvement strategies to better engage patients in SDOH screening and service utilization.

### 1.2. Literature Review

Our literature review indicates that the Chinese American community in Sunset Park, Brooklyn, faces many unmet needs. Structural factors, such as economic position, race, food access, and health system-level factors, may influence chronic disease outcomes, such as cardiovascular health [17] and cardiometabolic disease [18]. This social determinant of health gap among Chinese Americans may be due to cultural and linguistic misalignment in the healthcare setting [14]. In Sunset Park, Brooklyn, 21% of the U.S.-born and 8% of the immigrant population reported unmet health needs, which is defined as not receiving needed healthcare in the past year [15]. Additional research conducted at the NYU Langone Health Brooklyn Family Health Center, among 150 Mandarin-speaking Chinese Americans at risk for developing type 2 diabetes, reported an average of 2.5 SDOH barriers, with 82% of respondents reporting at least one SDOH barrier [19]. English literacy, racial discrimination, housing stability, childcare, and limited access to healthcare and social services were the top SDOH barriers identified. We sought to understand patient and provider perspectives on addressing unmet SDOH to aid in developing strategies to overcome these barriers.

## 2. Materials and Methods

### 2.1. Design

A qualitative study design was applied. Table 1 describes select key interview questions, which allow us to understand the SDOH services context and practice in a comprehensive way (guided by our integrated framework).

The interview questions outlined in Table 1 collected qualitative data on the SDOH implementation context to understand key factors in the healthcare system and service context that influence patient–provider communication and affect the relationship. Barriers and facilitators to SDOH-seeking behaviors were examined from both the patient and provider perspectives.

#### 2.1.1. Interview Sample

**Figure 2:**
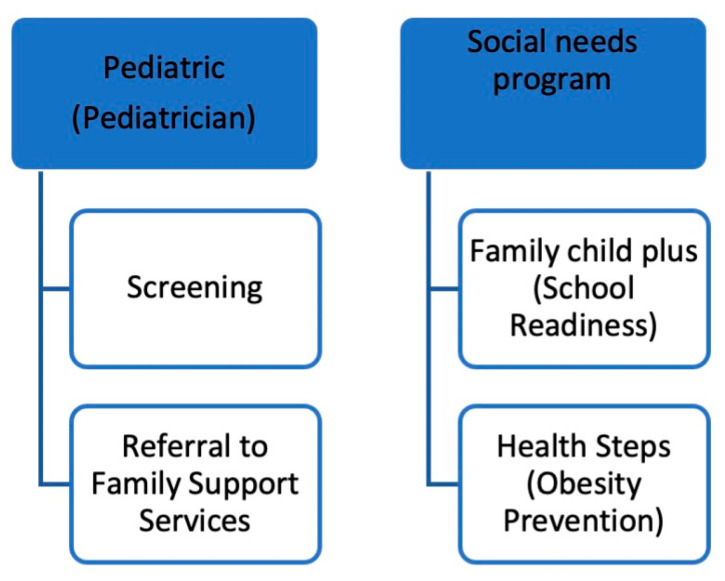
Caregiver Workflow for Social Determinants of Health Needs illustrates the provider workflow for patients with SDOH needs.

We recruited both providers (*n* = 8) and patients (*n* = 6) to gather representative perspectives as shown in Table 1. The term “representative perspective” was used, given that we recruited patients and providers across various clinical settings (e.g., family health centers) and social service offerings (e.g., Healthy Steps, Family Support Services) to encompass perspectives that were reflective of the sample population and representative of various perspectives. Three of the providers interviewed were in racial/linguistic affinity with the patient caregivers, which was representative of the Sunset Park community in Brooklyn. Providers included pediatricians, family practice doctors, and social service workers who serve a predominantly Chinese American patient population (see Figure 2). Patient caregivers of young children included caregivers who screened “Yes” to the following: self-identified as Chinese American, at least 18 years of age or older, the parent/caregiver of a child/children 3–7 years old, a current resident of Sunset Park, Brooklyn, and a fluent English, Mandarin, or Cantonese speaker. Providers were recruited via the snowball word-of-mouth method within the NYU Langone Health system, and caregiver participants were recruited via provider clinics. In-depth interviews with caregivers were conducted with Mandarin-speaking Chinese American caregivers were conducted. The interviews were offered in both Mandarin and Cantonese, but Cantonese speakers did not respond to study recruitment efforts.

#### 2.1.2. Data Analysis

The analysis was guided by the integrated Patient–Provider Partnership, Engagement, and Collaboration (PEC) framework, which emphasizes cognitive, affective/relational, and behavioral/communication domains in patient–provider interaction. Interviews began with healthcare professionals to identify system-level themes, which included categories related to healthcare, education, neighborhood and built environment, social and community context, and economic stability. These categories, based on Centers for Disease Control and Prevention classifications of social determinants of health, were used a priori to guide thematic development and were refined during the analytic process. The provider-identified system-level issues then prompted subsequent caregiver interviews, which were analyzed in relation to provider perspectives to capture the dynamic of patient–provider partnerships in addressing unmet needs.

Data analysis followed a structured six-phase process:

1. **Familiarization with the data:** All transcripts were read multiple times in their original language to ensure accuracy and depth of understanding.
2. **Generating initial codes:** A preliminary codebook was developed based on the interview guide, the interviewer’s notes, and emergent issues raised by participants as identified during repeated readings.
3. **Searching for themes:** Codes were clustered into broader categories reflecting cognitive, affective/relational, and behavioral/communication patterns, as well as contextual system-level factors.
4. **Reviewing themes:** Candidate themes were refined, checked against the dataset for internal consistency, and revised through team discussion.
5. **Defining and naming themes:** Final themes were clearly labeled to capture the essence of caregiver and provider experiences within the PEC domains and across system-level determinants.
6. **Configuring final analysis components:** To integrate perspectives systematically, final themes were organized into six components: Cognitive factors (knowledge, awareness, and provider understanding of cultural context). Affective/relational factors (trust, empathy, stigma, and interpersonal dynamics). Behavioral/communication factors (disclosure of needs, culturally concordant communication, and patient engagement). Contextual/structural factors (health system processes, staffing, intake workflows, and referral mechanisms). Broader systemic forces (economic instability, immigration stress, racism, and policy-level barriers affecting families).

Provider perspectives were essential in shaping the analysis. For example, providers highlighted barriers in economic stability (long waitlists for financial services, gaps in referral follow-up), healthcare access (reduced staffing, English-only intake forms, lack of EHR integration), education (caregiver anxiety about child developmental delays), neighborhood factors (housing instability and inadequate safe play areas), and social/community context (reluctance of caregivers to disclose needs due to stigma or cultural norms). These provider-informed themes provided context that enriched and guided the interpretation of caregiver experiences.

Two bilingual Mandarin–English-speaking researchers independently coded a subset of Mandarin transcripts in the original language to preserve cultural nuance. Codes were compared, and discrepancies were resolved through discussion, with a third researcher consulted as needed to reach consensus. The refined codebook was then applied to the full dataset. Themes were developed iteratively, with regular team debriefings to enhance reflexivity and ensure analytic rigor.

**Translation and Transcription Safeguards**

All caregiver interviews were conducted in Mandarin, transcribed verbatim, and coded in Mandarin prior to translation. Professional translation into English was completed by Vanam. Quality was safeguarded by a bilingual team member, a native Mandarin speaker, who cross-checked translations against the original transcripts to ensure accuracy of meaning. Provider interviews conducted in English were transcribed and verified by a research coordinator. These safeguards ensured that cultural nuance and meaning were preserved, a critical step in maintaining analytic validity in multilingual qualitative research.

Provider and caregiver interviews were conducted in Fall 2021, in the post-COVID-19 era that exacerbated SDOH needs, in an already low-resourced community. Interviews were transcribed and coded in Mandarin, and then translated into English by a vendor named Vanam. Codes were developed a priori based on the interviewer’s notes and analyzed by a Mandarin speaker. The application of the framework in Figure 1 towards provider perspectives on patient health-seeking behaviors leads to the following two categories: (1) Provider Office Workflow, and (2) Patient–provider interactions, which collectively affect patient engagement and collaboration strategies that affect their health-seeking behaviors.

#### 2.1.3. Ethical Considerations

In addition to obtaining IRB approval (study approval number: s21-00867), several steps were taken to handle ethical considerations around patient confidentiality and data protection, including informed consent. Provider interviews were a part of quality improvement, and participation in no way affected their employment. Providers did not speak of any one patient specifically or by name, but instead spoke on average regarding their experiences related to the questions asked.

The principal investigator (AC) ensured that this study was conducted in full conformity with Regulations for the Protection of Human Subjects of Research. Patients were not asked about their immigration status, birthplace, or area of origin during the interview. Providers described their perception of patient responses during interactions with the healthcare system due to perceived cultural stigma (e.g., perceived mistreatment due to the patient and provider having different national origins).

The informed consent process for eligible patients who expressed interest in the interview via RedCap included being contacted by a study staff member who spoke their preferred language during their preferred time. Patients were contacted by phone using WebEx Audio Conferencing Feature (no video, only the audio function) and were sent a secure RedCap link to review the consent form, using SendSafe. A staff member reviewed the consent form with the patient and paused during each section to check for understanding and answer questions. Patients were asked to summarize in their own words their understanding of what they were asked to do as part of their participation in the study. Patients were informed about the purpose of the study, what they were asked to do as part of their participation, the duration of their participation, risks/benefits, and compensation. They were informed that their participation was voluntary and that they could withdraw or discontinue at any time. Patients were asked to provide consent to audio record their interview. They were informed that if they did not consent to audio recording, they would not be able to participate in the interview. Once parents who indicated that they were interested in participating and agreed to participate, they were directed to provide e-consent in RedCap. If patients indicated that they were not comfortable or unable to provide consent via RedCap, they were offered the option to provide consent via paper. Once the e-consent or the paper consent was received, the interview was conducted with the parents.

Data protection included having all consent forms and documentation of consents stored in RedCap securely, with limited access to study personnel. All contact information was stored securely in RedCap, with limited access to the study PI (AC). Contact information was not connected to interview data. Any hard copies of contact cards or consent forms were stored in a locked cabinet with limited access to study staff only.

## 3. Results

Among the eight providers interviewed, four were Asian American, three were Hispanic, and one was Black. One provider was male, and the remaining were female. Three of the Asian American providers were in racial/linguistic affinity with their patient population and spoke Mandarin. All six of the patients/caregivers were Mandarin-speaking and were all female. All patients were caregivers of preschool-aged children, 3–5 years old, and were residents of Sunset Park, Brooklyn, a predominantly Chinese American and Hispanic immigrant community.

### 3.1. SDOH Services Processes from Provider Perspective

Provider interviews elucidated key areas of need, gaps, and strategies for addressing caregiver–patient social determinant of health needs (see Table 2). Screening service gaps related to service referrals and patients actually receiving services needed, centered around the following gaps, as outlined below:

• Short Staff and reduced family support staff/social workers post COVID-19 affected continuity of care to ensure SDOH needs were met, as well as relationship building among patients and providers. Also, the waitlist for social services may be up to two months. For instance, the provider quoted below stated,

*“Uh, for example we only know that we refer families with that families information and provide it with um the- shared all of the information with the family support service team, but we don’t know the status of um each case has been closed or not. Um the only way we were able to know is that we ask family, “Did you get support?” Normally family, if family don’t contact you, um they got support um quite probably within 24 h or something, but we get that feedback from family not from the staff member.”*

Quotes from the SNS team member

*“I think it would be helpful to have somebody from Healthy Steps here every day. We used to have someone here every day before COVID, and then of course COVID happened so. Because they’re the ones with all the resources and all the information for the programs, as far as the MAs, what we do is give the patient the form, have them fill it out, go in with the provider, they discuss with the provider, they discuss with the provider, then when they come out they’ll get the referral and the things that they need, but to actually have someone from the program...umm you know..hands on..someone who really needs it in the moment, you know..because we wouldn’t want anybody to fall out the loop….you know things happen in the process of things..not saying that it has happened, but if it’s something to be prevented to have someone there everyday to help the patient with these matters.”*

Quote from the medical assistant

*“I would say..umm. that relationship..umm.. trust the parents have with visitors allows for them to feel comfortable in sharing umm..or seeking support for the challenges they are experiencing. Wheras if it’s someone they don’t know, it would be a little bit challenging for them to share.. when they feel they can trust the person, they open up more.”*

Quote from the Healthy Steps provider

- Due to the immigration status, negative consequences, or not to bring “shame” on the family and disclose family struggles, caregivers did not complete the SDOH screeners completely. Patients ask for help within the family first. Additionally, fear of disclosing needs due to the immigration status may also be linked with cultural stigma or government mistrust. External trusted community partners outside the healthcare system may be engaged to assist patients with addressing an SDOH need. For instance, providers stated:

*“Um, so that’s one of the feedback that I got from the clinic staff, and um, and so yeah and that, that idea that sometimes there’s underreporting of needs and also our population, um our families are immigrant families, most of them, and there may also be some fear of reporting social needs and um because of their immigration status, but in general we haven’t had people refusing to fill it out or saying, “Why are you asking these questions?” So, people understand why we’re doing it.”.*

Quote from the SNS team member

*“So we do pair the families up with um someone who speaks the language and is also a cultural match. Um, but I’ve, I’ve never, like they’ve never, um, I think oftentimes what we hear is um from the Chinese speaking families is that they don’t need help right there. They don’t like to ask for help. So I guess, you know, in, in thinking about this, reflecting on it, um I think we have to find a better way to get them to feel comfortable to ask for help because there might be some needs, but they just don’t- like I think, I’m not sure if I’m wrong about this, but I feel that in the Chinese culture they, they don’t like to ask for help.”*

*Quote from Healthy Steps team member*

- Patient intake forms in written paper form in English were a limitation, given the time constraints of translation, which would take up time from the provider visit to provide care.

*“And then there’s a number that if you want to schedule a food pantry um they can call that number or if we check um that item for her, for that, for for for the participants, someone will also reach out to them to schedule a food pantry. Um, that they can grab the food, but um we don’t have very specific um program really geared towards scouting someone to uh support families filling different kind of forms in English and get finance support.”*

### 3.2. SDOH Services Processes from the Caregiver Perspective

Preliminary findings indicate caregivers had SDOH needs in mental health (e.g., anxiety, and childcare stress), food and financial security, but they tend not to utilize services because of beliefs of not wanting to burden others. Financial security was a prominent re-occurring theme that may have had trickle-down effects on food, housing security and active play time for the children, due to unemployment. Many mothers stayed at home while their husbands worked. Unfortunately, unemployment affected many of their husbands who worked in restaurants that shut down or were unemployed in other sectors. For instance, one mother stated,

“*My husband went out shopping once a week to buy all the things family needed. Prices were very high at that time. You could buy many things for over $100 before the pandemic, but at that time, it cost us almost $300 to $400 a week. Many things were in short supply, and prices were high.*”

Similarly, provider feedback aligned with caregiver needs for financial assistance in post post-COVID-19 timeframe, in an effort to meet caregivers’ needs. For instance, one provider stated, “I think just at the beginning of the pandemic, like 2020 when we asked those questions, when I ask a question about finance assistant or finance allowance, um they have lots of- they cannot- uh lots of follow up questions like, “who can help me to get different kind of allowance or different kind of finance- financial support?” Um, “can, can you refer someone directly to me um to address that um that question too, a little bit longer than the other ones?”

Another mother spoke of having to move due to increasing rent costs and needing a space/play area for her children. The mom stated,

“*My kids love to play with things that produce sound, such as banging on buckets and knocking on the floor. At that time, the downstairs complained about us, saying the kids were too noisy. Because of that and the fact that the rent was too high, we moved to…”*

When trying to ameliorate food, childcare/child activity, housing, or other social determinant needs, caregivers would rely upon their own social networks before going to providers. If caregivers seek out services, their experiences with providers are contingent on ethnic concordance, language, and cultural affinity to share needs and receptivity to available services.

Chinese American caregivers who utilized SDOH services within the healthcare system spoke of cultural discordance due to a lack of culturally appropriate offerings (e.g., the food pantry or languages spoken). One mother stated that the FHC staff helped her to register and receive food from the pantry, but she only went once because she thought that the food provided was more suited for Westerners. Since the food did not suit their cultural taste buds, she stopped going to the food pantry to avoid wasting any food. “*The food should go to people who need it. They also offered some sort of rice. They also passed out macaroni, but their children don’t usually eat macaroni or spaghetti,*” the mother stated. She described her family as having more traditional Chinese taste buds, so she did not want to receive the food from the pantry. Provider perspective of parents’ underutilization of the food pantry overlooked the contextual factor on the role of cultural appropriateness, so offerings are relevant and resonate with families. Cultural concordance was important for language, as well as nutrition offerings, as a bridge to help parents navigate available support.

Caregivers cautiously engaged in physical activity with their children, if any at all. In some instances, worries may trump opting to socialize or engage in physical activity, due to COVID-19 contagion fears. One caregiver describes her anxiety as follows,

“*I was actually okay. I guess it’s because of the pandemic, and I’ve been staying home for long. My mood will probably be better if I could go out somewhere to have a break. But at present, whenever I think about going out, my worries start to bother me. My cousins asked me if I was interested in joining them at the water park in the summer. But I didn’t dare to enter. Worries obsessed me at the very first moment when I thought about it. Things like clutters of people gathering, no face masks, and many more came to my mind.”*

Another mom stated,

*“My boy is well-behaved, and he wears masks properly. Some small children don’t want to wear masks. Maybe because my child has been staying at home for too long, he knows that once he wears a mask, he is going out to play. Therefore, he wears the mask well. We also disinfect in time after coming home. The situation is okay.”*

Limited physical activity and play due to isolation or staying indoors due to fear of contracting COVID-19 hampered children’s socialization, exercise, and possibly contributed to parenting stress. Social determinants of health may affect family health and wellbeing (e.g., financial, active play, and/or food security and parent stress) that could lead to long-term child development and physical health outcomes (e.g., weight status or cardiovascular health). Sustainable strategies and solutions are needed to overcome service gaps and meet social determinant health needs, especially in low-resourced immigrant communities.

## 4. Discussion

The Integrated Pediatric Health and Social Determinants of Health, within the focused context of the integrated PEC Framework in Figure 1, identifies gaps and potential opportunities for solutions via the following three areas: (1) Cognitive Strategies; (2) Affect and Relationship Strategies; and (3) Behavioral/Communication Strategies.

### 4.1. Cognitive Strategies

The patient screening process may introduce gaps through a lack of cultural cognizance. Cultural discordance of food offerings at the family health center, viewed by providers as a lack of engagement, may also be perceived by caregivers as not suiting their needs because the food was not representative of what the families ate. Provider understanding of culturally appropriate food offerings could address the food pantry utilization gap and better meet Chinese American families’ dietary needs.

In addition, language options in widely acceptable Chinese text, such as Simplified Chinese characters, in screening tools can be helpful for language appropriateness. Screening tool modalities may need to be adapted to best capture information about patients, ideally before patient care visits, to optimize workflow to allow greater time for patient care during visits. Evidence shows that Chinese Americans are avid users of technology [16]. Web-based or mobile app solutions translated into Simplified Chinese may be an optimal modality for capturing patient screening information before a visit. Non-tech-savvy users should also have the option for a Chinese-speaking staff member to walk them through the screener via a one-on-one process if needed as well.

Patient–provider information and referral service connection workflow may be hampered by cognitive gaps. Language concordance is critical when reaching out to low-income immigrant communities with a non-English language preference [20]. Language-based cultural affinity matching may be needed for patients to disclose social determinants of health needs, particularly around mental and emotional health concerns, such as immigration stress [21] and financial stress or caregiving stress [22]. Without cultural and language affinity matching for Chinese caregivers and providers/staff, racist social hierarchies will continue to perpetuate poor health effects among this population [23].

Cultural norms among Chinese American caregivers that may preclude them from wanting to “burden” others may lead them to share information if language-based and cultural affinity among patient–provider is evident. Conversely, providing knowledge gaps of patient social determinants of health needs hinders their ability to connect patients to referral services to meet their needs. Provider knowledge gaps may contribute to self-efficacy and confidence levels to address patient social determinant of health needs, as reflected by a national provider survey in which only 20% (*n* = 1000) reported feeling confident in their ability to address patient unmet social needs [24]. A holistic picture of a patient, inclusive of key social determinants of health factors, is needed for patient care.

### 4.2. Affect and Relationship Strategies

Patient-centered relationship strategies are essential for quality improvement in patient care. Patient interactions with staff or providers that may be perceived as rude/prejudiced, because of the judgment about the region of China where a person immigrated from, may contribute to negative affect. Affect may contribute to negative sentiment among patients, which may also affect patient-perceived healthcare quality. There is a grave need for staff cultural competency to understand that Chinese people are not a monolith. Strategies to address cultural competency include employment and retention strategies of ethnically diverse staff members, as well as cultural and structural competency training once hired, to raise cultural awareness and elucidate structural factors that contribute to stigma and health inequities [25,26]. Not all Chinese immigrants may speak the same Chinese dialect. Regional and cultural differences contribute to a person’s identity, and clinical staff interacting with Chinese American populations need a level of awareness of their cultural diversity, so they do not convey prejudice or bias among patient groups due to unconscious thoughts, feelings, or behaviors among providers that lead to negative affect that hamper the patient–provider relationship [27]. Provider bias introduced in the form of implicit bias, unbeknownst to their own ignorance of the needs and cultural sensitivity of Chinese American cultural groups, may affect the patient–provider partnership to address a health need. Providers may want to address patient needs but may be unaware of the language or cultural nuances of Chinese American patients to build rapport. Chinese Americans’ level of acculturation, such as first or second generation, may contribute to the level of willingness to receive social services, such as mental health, with less parental concern about burdening others.

### 4.3. Behavioral/Communications Strategies

Joo and Liu [10] identified five strengths of culturally tailored interventions: (1) Culturally respectful and patient-centered care; (2) Healthy Lifestyle promotion; (3) Increased Family and Community Supports; (4) Technology Use for Efficient and Timely care; and (5) Knowledge of Disease by Participants. Our study illuminated these same areas within the Chinese American community in Sunset Park, Brooklyn, by touching upon each of these five areas: (1) There is a need for patient–provider cultural and ethnic affinity/language matching to build trust and allow for disclosure of SDOH needs among caregivers to address respectful and patient-centered care. (2) Healthcare providers’ promotion of weight management as part of an obesity prevention program, and Family Child Plus, a school readiness program, illustrate healthy lifestyle promotion. (3) There is a strong family and community support network within the Chinese American community that they rely upon first, before seeking outside help. This interpersonal network is a strength to leverage communication dissemination strategies. However, heavy reliance on family networks may hamper the ability to address social determinant needs and other health-seeking behaviors if caregivers do not go outside of their ethnic enclaves [16], especially given the two-month waitlist reported by providers for health services. When thinking about the fourth strength of technology use for efficient and timely care, providers leverage WhatsApp for health promotion message dissemination among Chinese American caregivers. This strength of technology use and family connections could also be an opportunity area to have caregivers complete SDOH screeners in a Mandarin-translated digital mobile app or website before their medical appointment, to optimize care. Lastly, knowledge of disease by participants is exhibited by a need for greater patient/caregiver understanding of COVID-19 and how it affects their children. Immense provider misinformation about the spread of COVID-19 may thwart caregiver misconceptions that influence parenting behaviors around socialization, outdoor play, or school attendance.

## 5. Conclusions

Inclusion of both the patient/caregiver and provider perspectives is crucial for illuminating cognitive, affect/relationship, and behavioral/communication gaps. One person may not be aware of the other’s viewpoint or interpretation of a situation. Ultimately, by unveiling Chinese American caregiver and provider perspectives, we aimed to bridge caregivers and providers via inclusive family engagement strategies. Addressing social determinants of health among Chinese American immigrant populations is crucial for mitigating poor health outcomes in children and families. Multi-level community-engaged strategies are needed to alleviate the challenges facing this community. We hope our framework offers an integrated community-engaged strategy to overcome potential contextual, cultural, and consciousness-related barriers for Chinese American caregivers in meeting their social determinant of health needs.

### Significance and Implications of the Study

Our framework is the only one taking an inclusive approach to addressing the social determinants of health needs of Chinese Americans. Existing literature has harnessed cultural sensitivity and language-based approaches to engaging Chinese American immigrants in health interventions [28,29], but may be limited by the lack of knowledge of cultural nuances and social contextual factors that influence caregiver health-seeking behaviors [30]. Having an understanding of the community’s organizational, cultural, and contextual factors is pivotal for future implementation of health behavior programs or intervention strategies targeting Chinese Americans, as they especially relate to adoption, adaptation, and sustainability implementation science outcomes [30,31,32]. In addition, future research should also consider a larger sample size to bolster power and carry out this work. Lastly, our examination of both patient and provider perspectives may have shed light on unknown harm, such as provider bias, a critical factor in community-engaged research, heightened in the COVID-19 era [33], that has a known effect on the physical and mental health of Chinese Americans [30]. Elucidating patient and provider perspectives sheds light on culturally and racially/ethnically affirming strategies that could reduce harm and optimize caregiver experiences and care to provide for their families.

## Figures and Tables

**Table 1:** Provider Perspective on Family Health Center and Social Need Service Context.

FHC service context (Pediatric setting)	**Provider Care Team in Pediatric Setting:** Screening + (social needs programs include: Healthy Steps (an obesity prevention program), ParentChild + (a home visiting program to support child developmental milestones), and Family Support Services (FSS) connect to community resources to address needs (e.g., childcare, transportation, food/financial security, ESL classes).
Interview sample	*n* = 2 Pediatrician + 6 SNS staff
Sample interview questions	Q1. *Describe the processes* for screening a patient *for social needs (i.e., food, childcare, housing) when they arrive for a clinical visit.* Q2. *What is the process for referring a patient who screens positive for social needs?*
Select thematic responses	Q1 Patient Screening process included: • Counselors/community health workers/medical assistants who speak the patient’s language conduct screening and refer the patient to Family Support Services (FSS) for an identified SDOH need to support the patient when applying for public assistance (e.g., food stamps/rent assistance). Q2 Referral process • Medical provider refers patient directly to the Healthy Steps team member if a staff person is present. • All referrals from the SDOH screener are sent via EPIC to FSS.

**Table 2:** Patient Perspective for Access and Social Need Utilization.

Interview Sample	6 Chinese American Caregivers
Sample Patient Questions	Where do you go for your first point of contact for these needs (e.g., food, housing, childcare, financial assistance)? Is there a trusted person in your community that you might reach out to first?
Select Thematic Responses	Caregivers have a support network of family, friends, and organizations that can be contacted for resources, but display a willingness to only reach out if necessary to avoid burdening others. Resources recommended by organizations may be challenging to access (e.g., long wait time) and may deter them from utilizing them.

## Data Availability

The data presented in this study are available on request from the corresponding author. The data are not publicly available due to privacy.

## Abbreviations

The following abbreviations are used in this manuscript:

SDOH	Social determinants of health
CA	Chinese Americans
PEC	Partnership, Engagement, and Collaboration
FHC	Family Health Centers
FSS	Family Support Services
SNS	Social Need Service
